# Histological and Immunofluorescence Study of Discal Ligaments in Human Temporomandibular Joint

**DOI:** 10.3390/jfmk5040090

**Published:** 2020-12-08

**Authors:** Michele Runci Anastasi, Antonio Centofanti, Alba Arco, Giovanna Vermiglio, Fabiana Nicita, Giuseppe Santoro, Piero Cascone, Giuseppe Pio Anastasi, Giuseppina Rizzo, Giuseppina Cutroneo

**Affiliations:** 1 I.R.C.C.S. (Istituto di Ricovero e Cura a Carattere Scientifico) Centro Neurolesi “Bonino Pulejo”, 98124 Messina, Italy; miki_runci@hotmail.it; 2 Department of Biomedical and Dental Science and Morphofunctional Imaging, University of Messina, 98122 Messina, Italy; centofantia@unime.it (A.C.); alba.arco@unime.it (A.A.); fabin92@hotmail.it (F.N.); giuseppe.santoro@unime.it (G.S.); anastasi.giuseppe@unime.it (G.P.A.); giuseppina.rizzo@unime.it (G.R.); 3 Department of Maxillo-Facial Surgery, University of Roma La Sapienza, 00185 Rome, Italy; piero.cascone@uniroma1.it; 4 Department of Clinic and Experimental Medicine, University of Messina, 98122 Messina, Italy; giuseppina.cutroneo@unime.it

**Keywords:** human TMJ, discal ligaments, histological techniques, immunofluorescence

## Abstract

The temporomandibular joint (TMJ) is a bilateral synovial articulation stabilized by several anatomical structures such as ligaments. The existence of articular capsule reinforcement structures have been described in the lateral and medial sides of disc which have been defined as collateral ligaments, lateral and medial. Despite that, some macroscopic observations support that these collateral ligaments do not belong to the articular capsule but they belong to the disc. By that, the aim of the present work was to evaluate morphological aspects of TMJ from cadaveric frozen heads by histological and immunofluorescence techniques in order to verify the origin and insertion of lateral and medial collateral ligaments. Results show that both lateral and medial ligaments origin from the disc and insert directly to the articular cartilage of mandibula condyle. These data open a new approach in the study of human TMJ.

## 1. Introduction

The temporomandibular joint (TMJ) is a bilateral synovial articulation between condylar process of the mandibular ramus [1] and the mandibular fossa of the temporal bone. This joint it is also made up of thin fibro-cartilage articular disc, interposed between the articular items, and of a loose joint capsule that surrounds the lateral sides of the joint [2,3,4].

The disc, together with the articular capsule, the temporomandibular ligament, the accessory ligaments, and the muscles play a key role in joint stabilization [5]. Several diseases could compromise the stability of TMJ joint bringing to macro- and microscopical disc pathological features with the consequential onset of clinical alterations as articular dislocation [6,7,8,9,10].

The TMJ disc is a fibrocartilage tissue, biconcave and elliptical in shape with two faces, superior and inferior, two margins, medial and lateral, and two extremities, anterior and posterior [11,12]. Its particular shape allows a smooth articulation between the mandibular condyle and the articular eminence and increases the contact area between opposing articulating surfaces, distributing lower stresses to a larger surface area in the joint [13,14,15]. The anterior portion of the articular disc attaches superiorly to the articular eminence by bending with the joint capsule and inferiorly to the anterior condyle and the upper area of the lateral pterygoid muscle. Posteriorly, the disc attaches superiorly to the temporal bone and inferiorly to the posterior condyle.

Laterally and medially, the disc attachments blend into the joint capsule near its attachment to the condylar head [16,17,18]. Some reports have described the existence of articular capsule reinforcement structures in the lateral sides of disc which have been defined as collateral ligaments, lateral and medial [19,20]. Despite that, the existence of both collateral ligaments is not unanimously accepted, and Bravetti et al. [21] have characterized the existence of the collateral lateral ligament but not the collateral medial one by histological evaluation on human TMJ.

Although in the literature the collateral ligaments have been described as reinforcement structures of capsule, other sources suggest that the collateral ligaments would be intrinsic ligaments of discs since they would originate directly from the lateral sides of the disc [20,22]. No more data demonstrating the existence of these “discal” ligaments exist in the literature and they remain described only through anatomical drawings.

The discordances in anatomical descriptions of human TMJ depend on the fact that the macroscopic investigations could not be sufficient for a correct identification and description of all morphological structures, especially for the study of human TMJ which is a complex structure hidden in the base of the cranium.

A microscopic approach in the study of human TMJ would allow for more morpho-functional information. Despite that, microscopic studies are limited by the difficulty of finding human cadaveric TMJ [23,24]; in fact, most histological studies are performed on animal models [25,26].

Based both on the controversial anatomic descriptions of human TMJ and on the lack of microscopic investigations, the aim of the present study was to evaluate morphological aspects of TMJ from cadaveric frozen heads by histological and immunofluorescence techniques in order to verify the origin and insertion of lateral and medial collateral ligaments.

## 2. Materials and Methods

The study was performed on intact block specimens of temporomandibular joint obtained from 10 heads of human cadavers (6 male and 4 female, 65 ± 3 years old) preserved in the anatomical museum of Department of Biomedical and Dental Sciences and Morpho-functional Images of University of Messina, Italy. The anatomical samples of TMJ were selected according to SCAPINO criteria [27]: smooth articular surfaces, articular disc without perforations, its posterior band situated above or only slightly anterior to the summit of the condyle in all regions, and no signs of disc displacement or temporomandibular disorder [28]. Cases with bony pathology of the temporo-mandibular joint seen on radiographs were excluded.

The macro-anatomical dissection of the specimens was carried out in sagittal planes with the vision of the articular eminence, TMJ disc in antero-posterior view, condylar bone portion, and retro-discal tissue. Subsequently, we analyzed the macroanatomical dissections of the samples on the coronal plane. We obtained serial histological sections of lateral and medial sides of the articular disc in the antero-posterior direction. These disc portions and their relationship with the mandibular condyle were assessed by means of the light microscopy and immunofluorescence.

### 2.1. Light Microscopy

After defrosting, the specimens were fixed overnight in 4% paraformaldehyde in 0.05 M phosphate buffer at 4 °C, dehydrated in ethanol and infiltrated with Technovit9100. Sections of 7 μm thickness were cut with a microtome Leica RM 2125RT (Leica Biosystem, Nussloch, Germany) and were stained with hematoxylin and eosin method [29,30]. The sections were examined and photographed with a light microscope Eclipse Ci-L (Nikon Corporation, Tokyo, Japan).

### 2.2. Immunofluorescence

The same section obtained for light microscopy were used to perform immunofluorescence reactions. The sections were placed on glass slides that were coated with 0.5% gelatin and 0.005 chromium potassium sulphate. To block nonspecific binding sites and to permeabilize the membranes, the sections were preincubated with 1% bovine serum albumin (BSA), 0.3% triton X-100 in PBS for 15 min at room temperature [31,32]. Finally, the sections were incubated with primary antibodies. We used the following primary monoclonal in mouse antibodies: anti-collagen type I (Sigma Aldrich, St. Louis, MO, USA) dilution range 1:1000; anti-elastin (Santa Cruz Biotechnology Inc., Santa Cruz, CA, USA) dilution range 1:50.

Primary antibodies were detected using Texas-Red conjugated IgG anti-mouse (Jackosn ImmunoResearch Laboratories, Inc., West Grove, PA, USA) and Fluorescein Isothiocyanate (FITC) (Jackosn ImmunoResearch, Inc., West Grove, PA, USA) both at dilution range 1:100.

The sections were analyzed and images acquired using a Zeiss LSM 5 Duo (Carl Zeiss, Iena, Germany) confocal laser scanning microscope [33,34,35]. All images were digitalized at the resolution of 8 bits into an array of 2048 × 2048 pixels. Optical sections of fluorescence specimens were obtained using a HeNe laser (wavelength = 543 nm) and an Argon laser (wavelength = 458 nm) at a 762-s scanning speed with up to eight averages; 1.50 μm thick sections of fluorescence specimens were obtained using a pinhole of 250. Contrast and brightness were established by examining the most brightly labeled pixels and choosing the settings that allowed clear visualization of the structural details while keeping the pixel intensity at its highest (200). Each image was acquired within 62 s, in order to minimize the photodegradation [36,37,38,39,40]. For image analysis, we used the function called “splitting”, showing individual channels. Digital images were cropped, and figure montages were prepared using Adobe Photoshop 7.0 (Adobe System, Palo Alto, CA, USA).

## 3. Results and Discussion

Ligaments of temporomandibular joint are represented by the lateral or temporomandibular ligament, the anterior ligament of the malleus and the sphenomandibular, stylomandibular, and pterygomandibular ones [11,41,42,43,44].

Moreover, there are two ligaments originating from the articular capsule that would play the role of lateral stabilization of the disc to the bone surface: these connective structures are named collateral lateral and collateral medial ligaments [2,19,20].

The existence of both collateral ligaments is controversial since the data present in the literature are discordant. Some authors have described the collateral lateral by radiological and histological means but the collateral medial one has not been found by histological investigation [21].

Although it has been suggested that the collateral ligaments represent reinforcement structures of the capsule, some reports support that the collateral ligaments would belong to the disc and not to the capsule since their fibers would originate from the disc; these “discal” ligaments have been shown only by anatomical drawing [2,20]. This description arises from intraoperative macroscopic observation and no microscopic morphological data are available.

**Figure 1:**
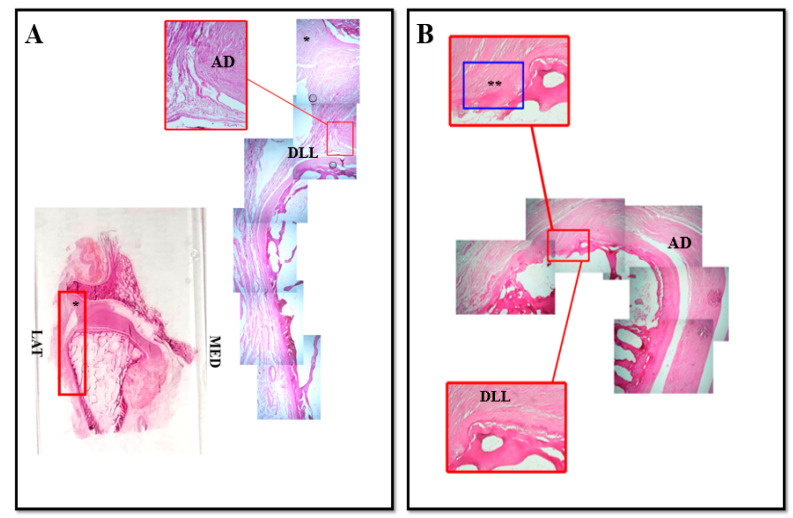
Compound panel of Hematoxylin-Eosin staining of human TMJ, lateral side (red box). Sections were reconstructed in order to observe a wide microscopic field at the level of the lateral side of TMJ. It is possible to observe the existence of an anatomical structure that made up of nondissociable fibers that seems to give life to two connective bundles: the first one originates from the lateral region of the disc and, running upwards, it inserts on the capsule ((**A**), asterisk); the second one originates from the lateral region of the disc and it runs downwards inserting on the superficial layer of the articular cartilage of the condyle ((**A**), DLL). In our opinion this second structure corresponds to the discal lateral ligament; in (**B**) pictures show the direct insertion of discal lateral ligament on the superficial layer of mandibular condyle cartilage surface (double asterisk). AD: articular disc; DLL: discal lateral ligament; LAT: lateral; MED: medial.

**Figure 2:**
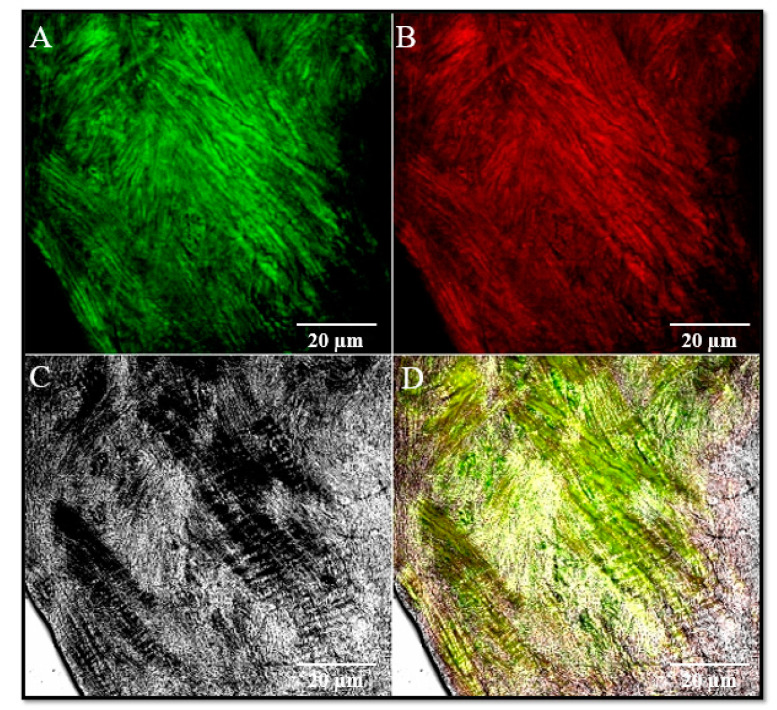
Double localization immunofluorescence reaction performed on discal lateral ligament using antibody against Collagen I (**A**), green channel and Elastin (**B**), red channel. Pictures show an intense fluorescence pattern for both proteins (**A**,**B**). (**C**), transmitted light (TL); (**D**), the yellow fluorescence depends on the merge of green and red channels. Magnification: 40×.

Our results obtained by coronal section of human TMJ show all the classical components of the joint: the fibrous capsule in the lateral sides of the joint, the disc and the articular surfaces represented by temporal bone fossa and mandibular condyle (Figure 1 and Figure 2).

It is possible to observe in the lateral side of the articular disc (Figure 1) the existence of an anatomical structure made up of nondissociable fibers that seems to give life to two connective bundles: the first one originates from the lateral region of the disc and, running upwards, it inserts on the capsule; the second one originates from the lateral region of the disc and it runs downwards inserting on the superficial layer of the articular cartilage of the condyle (Figure 1B, asterisk).

In our opinion, this structure corresponds to the lateral collateral ligament that, on the basis of our results, it would not belong to the capsule but it would be an anatomical structure that originates directly from the articular disc. By that we can name this ligament “lateral discal ligament” (Figure 1A, DLL). The insertion of this ligament on the articular cartilage of mandibular condyle surface reinforces the idea that this ligament plays a key mechanical role in strongly stabilizing the disc to the mandibular condyle.

This hypothesis is supported by immunofluorescence results that show in the lateral region of the disc and in the lateral discal ligament the expression of both collagen IV and elastin, mainly components of the ligamentous fibrous connective tissue (Figure 2).

**Figure 3:**
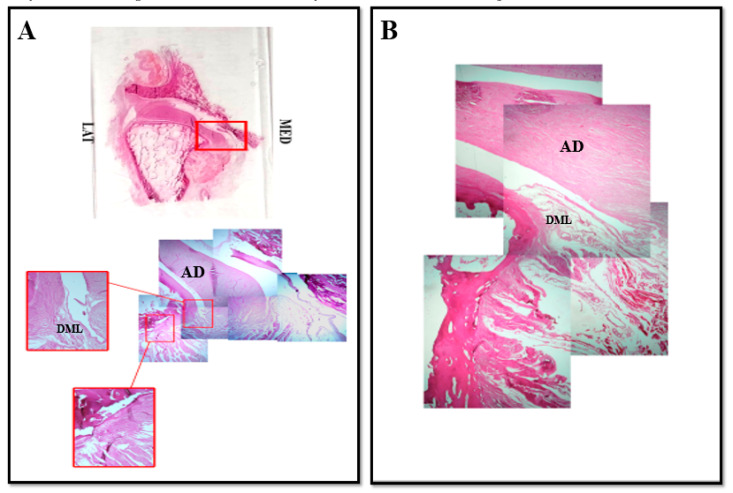
Compound panel of Hematoxylin-Eosin staining of human TMJ, medial side (red box). Sections were reconstructed in order to observe a wide microscopic field at the level of the medial side of TMJ. It is possible to observe the existence of an anatomical structure made up of loose fibers that originate from lateral side of articular disc and insert on articular cartilage of mandibular condyle in the medial side (**A**), DML. In our opinion this structure corresponds to the medial lateral ligament; in (**B**) pictures show the direct insertion of discal lateral ligament on the superficial layer of mandibular condyle cartilage surface. AD: articular disc; DML: discal medial ligament.

About the medial region of the TMJ (Figure 3), our findings show that it is not possible to identify the same connective structure corresponding to the capsule that we observed in the lateral side. That is in accordance with previous report [21], supporting that at medial level the TMJ is not anatomically closed by articular capsule and delimited by a medial collateral ligament.

**Figure 4:**
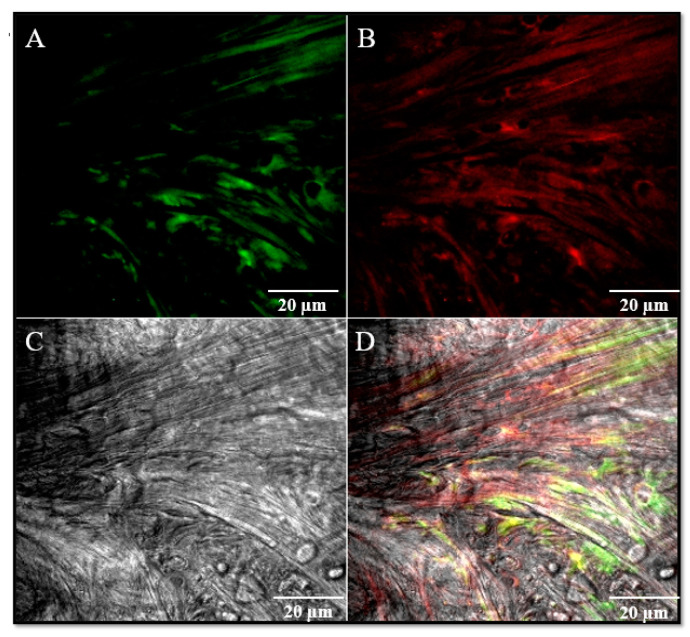
Double localization immunofluorescence reaction performed on discal lateral ligament using antibody against Collagen I (**A**), green channel and Elastin (**B**), red channel. Pictures show an intense fluorescence pattern for both proteins (**A**,**B**). (**C**) transmitted light (TL); (**D**) the yellow fluorescence depends on the merge of green and red channels. Magnification: 40×.

Despite that, it is possible to observe the existence of fibers that originate from the postero-medial portion of the disc and they fold into a loop to then insert to the articular cartilage of the condyle and run downwards along the periosteum (Figure 3). This structure would correspond to the collateral medial ligament even if this ligament does not have the same morphology observed for the lateral collateral ligament. By that, we suggest that also this structure could represent an intrinsic “discal” medial ligament that ensures a tight mechanical anchorage of the disc on the mandibular condyle surface at medial level, even more so that no other connective structures that close and delimit the medial region of the TMJ have been identified. We could name this ligament “medial discal ligament”. All these data are supported by immunofluorescence results that have shown in the medial side of the disc and in the medial discal ligament the expression of both collagen IV and elastin (Figure 4).

Our results describe for the first time, at microscopic level, the origin and the insertion of collateral ligaments; until now they were considered to belong to the capsule but our data show that these collateral ligaments would belong to the disc since their fibers originate directly from the lateral and medial regions of the articular disc. Moreover, the insertion of these ligaments on the articular cartilage of the condyle also suggest that they play a mechanical role in order to maintain disc stabilization on the condyle surface.

## 4. Conclusions

The demonstration of the existence of intrinsic “discal” ligaments in human TMJ could open a new approach in the study of the anatomy of human TMJ. That can also have clinical and surgical impact as damages at discal ligaments level could alter the stability of the joint determining morpho-functional alterations.
